# Improving Recycled Poly(lactic Acid) Biopolymer Properties by Chain Extension Using Block Copolymers Synthesized by Nitroxide-Mediated Polymerization (NMP)

**DOI:** 10.3390/polym13162791

**Published:** 2021-08-19

**Authors:** Juan José Benvenuta-Tapia, Pascale Champagne, José Alfredo Tenorio-López, Eduardo Vivaldo-Lima, Ramiro Guerrero-Santos

**Affiliations:** 1 Facultad de Ciencias Químicas, Universidad Veracruzana, Coatzacoalcos 96535, Veracruz, Mexico; altenorio@uv.mx; 2 Institut National de la Recherche Scientifique, Centre Eau Terre Environnement, Québec City, QC G1K 9A9, Canada; 3 Facultad de Química, Departamento de Ingeniería Química, Universidad Nacional Autónoma de México, Ciudad de México 04510, Mexico; 4 Polymer Synthesis Department, Centro de Investigación en Química Aplicada (CIQA), Blvd Enrique Reyna Hermosillo, Saltillo 25294, Coahuila, Mexico

**Keywords:** biopolyester, poly(lactic acid), chain extenders, polymer recycling, biopolymers, nitroxide-mediated polymerization

## Abstract

The aim of this contribution is to assess the use poly(styrene-*co*-glycidyl methacrylate-*b*-styrene) copolymers synthesized by nitroxide mediated polymerization (NMP) as chain extenders in the recycling of poly(lactic acid) biopolyester. Concisely, the addition of such block copolymers during the melt processing of recycled poly(lactic acid) (rPLA) leads to important increases in the viscosity average molecular weight of modified polymeric materials. Molar masses increase from 31,000 g/mol for rPLA to 48,000 g mol^−1^ for the resulting rPLA/copolymer blends (bPLA). Fortuitously, this last value is nearly the same as the one for pristine PLA, which constitutes a first piece of evidence of the molar mass increase of the recycled biopolymer. Thermograms of chain extended rPLA show significant decreases in cold crystallization temperature and higher crystallinity degrees due to the chain extension process using NMP-synthesized copolymers. It was found that increasing epoxide content in the NMP-synthesized copolymers leads to increased degrees of crystallinity and lower cold crystallization temperatures. The rheological appraisal has shown that the addition of NMP synthesized copolymers markedly increases complex viscosity and elastic modulus of rPLA. Our results indicate that P(S-co-GMA)-*b*-S) copolymers act as efficient chain extenders of rPLA, likely due to the reaction between the epoxy groups present in P(S-co-GMA)-b-PS and the carboxyl acid groups present in rPLA. This reaction positively affects viscometric molar mass of PLA and its performance.

## 1. Introduction

Poly(lactic acid) (PLA) has attracted much attention in recent years as an environmentally biodegradable polymer which may serve as replacement of conventional petrochemical-based polymers [1]. PLA is generally synthesized by ring-opening polymerization of lactide, which is the lactone cyclic di-ester derived from lactic acid (2-hydroxy propionic acid) [2].

PLA has good transparency, moderate toughness, barrier properties and biocompatibility, which lead to its wide applicability [3,4]. As stated earlier, such features make it suitable for substitution of some petroleum-based polymers. Still, what really stands out with PLA is its biodegradability and the fact that it is completely derived from renewable resources. That is why this biopolymer has attracted substantial attention in recent years. All in all, it is an outstanding alternative to help overcoming the plastic pollution observed in the last decades [5,6].

Accordingly, as for now, PLA and their copolymers are a unique exceptional alternative to conventional polymers in commodity applications, such as packaging, especially for food products, due to its good processability and low environmental impact [7,8,9,10,11,12]. Furthermore, PLA has been widely investigated for use in biomedical applications, such as tissue engineering scaffold [13], bone grafting and drug delivery systems [14], because of its biocompatibility with human tissues. It is also important for implants that are expected to remain in contact with human tissues for a long time. For that reason, this biopolymer has been also a subject for meticulous safety evaluations [15].

All things considered, while PLA is a relatively new material in the plastics industry, it has the potential to become widely used in a broad range of applications. The inappropriate disposal of residues combined with the rather slow biodegradation rate in nature, may lead to the proliferation of PLA waste [15]. Therefore, it is important to timely search for recycling routes for this valuable material and develop innovative applications for the recycled products [16].

Mechanical recycling of PLA by melt reprocessing has gained importance due to its simplicity, low investment requirements, and minimal adverse environmental impact [17,18,19]. However, a common problem observed during PLA recycling by this thermo-mechanical reprocessing is that polymer molecules undergo thermal and oxidative degradation, which leads to molar mass reduction, lost of mechanical properties, and poor performance of recycled materials (rPLA) [20]. Consequently, there is an ineludible need to develop a suitable technology to avoid PLA degradation along with maintaining the biopolymer properties, in order to make it suitable for different applications.

The use of chain extenders to overcome the deterioration of properties and performance of polyesters such as PLA and PET during recycling by melt processing, has been described to be a very good alternative [21,22,23,24]. Chain extenders are usually multifunctional small molecules that contain functional groups with the ability to react with the polymer end groups, PLA in our case. That is, the chain extender reacts with PLA end groups to reconnect the polymer chains broken during processing, thus leading to polymer molecules with higher molar masses and restored properties [25].

Some of the chain extenders used for the reprocessing of PET or PLA are toxic chemicals which may constrain the niche applications of PLA. Examples of such chain extenders are bifunctional 1,6-diisocyanatohexane [23] and tris(nonyl phenyl) phosphite (TNPP) [24]. TNPP has been used to increase the stability of PLA during melt processing [24], but its use is discouraged mainly because the nonyl-phenol formed during processing is known as an endocrine-disrupting chemical, and also a tumoral agent [26].

On the other hand, block copolymers containing epoxide groups are able to react with functional groups of the polymer being processed, and also render softer materials. These features enhance the range of processability, stability of post-consumer polyesters, and minor toxic effects can be anticipated [21]. Other functional polymers can be used as efficient chain extenders, for instance those synthesized by conventional free-radical copolymerization (FRP) [21,22,27,28,29,30,31,32], providing improved thermal stability and enhanced mechanical properties of the recycled polymer [33,34,35,36,37].

Although polymer synthesis by FRP has the advantage of requiring undemanding reaction conditions and the ability to polymerize a large number of monomers, its main drawback is poor control of both molar mass distributions, which are usually broad, and polymer microstructure (e.g., copolymer composition heterogeneities) of the produced materials [38]. The above-mentioned features can be transferred to the extended polymer, i.e., the heterogeneities produced by FRP can be exacerbated by the aleatory reaction with the polymer to be extended. The resulting blends may have even wider molar mass distributions, which may have undesirable effects on fluidity during processing of final items. Therefore, we decided to use reversible deactivation radical polymerization (RDRP) techniques to get a better control of molar mass heterogeneity of the polyesters thermally blended with the copolymer extenders synthesized herein. As it is known, RDRP allows the synthesis of polymers and copolymers with controlled microstructures. In the case of copolymers, molar mass and copolymer composition distributions of simultaneously reduced heterogeneities can be targeted [39].

In previous contributions from our group, we reported the chain extension of recycled PET (rPET) by the addition of copolymers synthesized by reversible addition-fragmentation transfer (RAFT), a type of RDRP technique [40,41]. The application of these RAFT-synthesized copolymers during the processing of rPET considerably improved the molar mass and rheological properties of rPET. However, a characteristic odor and yellowing appear during the confinement of products in the presence of oxygen, due to the degradation of sulfur-containing fragments present in this type of polymers.

For this reason, nitroxide mediated polymerization (NMP), another distinctive case of RDRP, was employed in this investigation. NMP is one of the most important RDRP techniques and it is relatively simple to carry out since it only involves heat and the appropriate nitroxide controller to initiate polymerization, and it does not need extensive post-polymerization processes to eliminate remaining catalysts and other chemicals or metallic residues [42]. Unlike the RAFT technique, NMP does not require to remove sulfur odors from the chain transfer agent [38].

Initially, NMP was only applicable to styrenic monomers using 2,2,6,6-tetra-methyl-1-piperidinyloxy (TEMPO) [43,44]. A second-generation of nitroxides which included the so-called SG1 (N-(2-methylpropyl)-N-(1-diethylphosphono-2,2-dimethylpropyl)-N-oxyl), commercially available as “BlocBuilder” (Arkema), extended the use of NMP to acrylates and acrylamides [45,46].

Unfortunately, SG1 is not effective for the copolymerization of monomer mixtures containing GMA since a side-reaction between the epoxy groups contained in GMA and carboxylic acid groups present in SG1 is possible. This reaction may lead to the production of branched, insoluble products. However, if SG1 is protected by introducing a succinimidyl ester moiety into it (NHS-SG1), then the nitroxide-mediated copolymerization of GMA and styrene over a wide range of comonomer compositions is possible [47,48].

In this contribution, the synthesis of reactive poly(styrene-co-glycidyl methacrylate)-block-polystyrene (P(S-co-GMA)-b-PS) copolymers with controlled block lengths and high content of epoxy functional groups, by NMP, for a more uniform and efficient performance during PLA recycling, is reported. To the best of our knowledge, there are no other reports available on-chain extension of recycled PLA using this type of block copolymers as chain extenders. These copolymers are evaluated as potential non-toxic chain extenders for production of rPLA. As stated earlier, the epoxy groups present in the synthesized reactive copolymers can react with the carboxylic acid functional groups present in recycled PLA. The PS block provides adequate processing and thermal properties to the P(S-co-GMA)-b-PS copolymer.

PLA was subjected to thermo-mechanical degradation by melt extrusion in two extrusion cycles, in order to simulate the recycling process.

The molar mass of the extruded biopolymer decreased 25% in the first cycle and reached 55% of reduction after the second extrusion. The mechanical properties also worsened. Other groups have reported similar procedures and results regarding the emulation of recycling by two processing steps in screw extruders [17].

This rPLA was then processed by twin extrusion technology with the addition of different amounts of NMP-synthesized copolymer as chain extender. The performance and properties of the resulting rPLA modified with our copolymers was assessed through detailed characterization, including the measurement of physical, thermal, and rheological properties.

## 2. Materials and Methods

### 2.1. Chemical Reagents and Polymers

GMA (97%) and styrene (S) (99%) were purchased from Aldrich (Saint Louis, MO, USA). They were purified by passing them through columns packed with inhibitor remover and then stored in a sealed flask under a head of nitrogen in a refrigerator, until needed.

N-(2-Methylpropyl)-N-(1-diethylphosphono-2, 2-dimethylpropyl)-*O*-(2-carboxylprop-2-yl) hydroxylamine (99%, SG1, Arkema) was used as received. N-Hydroxysuccinimide (98%) and N,N′-dicyclohexylcarbodiimide (DCC, 99%), both from Aldrich (Saint Louis, MO, USA), were reacted with SG1 to produce the succinimidyl ester terminated alkoxyamine (NHS-SG1), according to literature procedures [47,48].

l-lactide was purchased from Purac (Gorinchem, Netherlands) and was used for polymerization after purification by recrystallization from ethyl acetate solution followed by 3 h of drying under vacuum. Ethyl acetate, stannous octoate Sn(Oct)_2_, (95%), and 1-dodecanol (98%) were purchased from Aldrich (Saint Louis, MO, USA), and were used as received. 1-Dodecanol was purified by distillation under reduced pressure before use. All reagents used were analytical grade.

### 2.2. Synthesis of PLA

**Figure 1:**
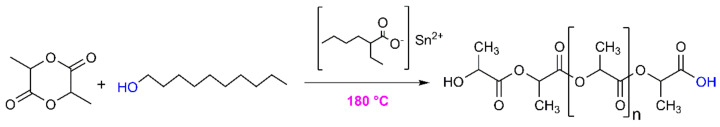
Scheme for the synthesis of PLA by ring-opening polymerization.

PLA was synthesized by ring-opening polymerization of l-lactide in bulk at 180 °C for 2 h under a nitrogen atmosphere using stannous octoate (0.20 mol%) as catalyst and 1-dodecanol (1.60 mol%) as cocatalyst of the polymerization system (see Figure 1). The resulting biopolyester was then granulated before drying in a vacuum oven at 100 °C for 6 h to remove the unreacted monomer. The obtained PLA showed a number average molar mass of 70,000 g mol^−1^.

### 2.3. Synthesis of Poly(S-co-GMA) Copolymers by NMP

Copolymerizations of S and GMA were carried out in bulk at 105 °C in a 1-L high-pressure stainless steel jacketed reactor (Parr Model 4523, Moline, IL, USA) with temperature control, as well as pressure and stirring sensors. The reactor was purged twice with high-purity nitrogen to maintain an inert gas atmosphere. It was then charged with calculated amounts of glycidyl methacrylate and styrene, ranging from 35–55 mol% GMA, followed by the corresponding amounts of N-hydroxysuccinimidyl-functionalized NMP controller (NHS-SG1, Aldrich, Saint Louis, MO, USA). A theoretical number average molar mass (M_n,th_) of 30,000 g mol^−1^ was targeted for all copolymers. Total reaction time was 4 h. The polymer product was removed through a discharge line leading to a sampling vessel that was cooled to stop the polymerization. The resulting copolymers were re-precipitated in hexane, decanted and dried overnight in a vacuum oven at 60 °C for 24 h to obtain the final purified copolymers. The detailed experimental conditions are provided in Table 1.

#### Chain Extension Experiments

The P(S-co-GMA) copolymers synthesized in the previous section were withdrawn from the reactor for purification purposes. Once the purification process was completed, they were used as macroinitiators in chain-extension polymerizations by the addition of 30 wt% purified styrene monomer to the reaction mixtures containing macroinitiators dissolved in toluene under magnetic stirring. After purging with nitrogen at room temperature for 30 min, the temperature was increased to 105 °C to initiate the chain extension reaction. The block polymerization step proceeded for 3 h. At the conclusion of the polymerization after cooling the mixture to T < 40 °C, the contents were purified by precipitation and vacuum dried overnight in a vacuum oven at 60 °C to remove any remaining solvents or unreacted volatile monomers (styrene). Tree P(S-co-GMA)-b-PS copolymers having approximately 30, 35 and 45 mol% GMA, denoted as P(S-co-GMA)-b-PS-1, P(S-co-GMA)-b-PS-2, and P(S-co-GMA)-b-PS-3 respectively, were obtained. A theoretical Mn of 40,000 g mol^−1^ was targeted for all P(S-co-GMA)-b-PS copolymers.

### 2.4. Characterization of P(S-co-GMA) and P(S-co-GMA)-b-PS Copolymers

Gel permeation chromatography (GPC 515, Waters, Milford, MA, USA) was used to determine number- and weight-average molar masses (M_n_ and M_w_) and dispersity (Ð) of the synthesized copolymers. The analyses were carried out using a Waters 1515 gel permeation chromatograph equipped with HR 1, HR 3, and HR 4 columns, thus covering a molecular weight range of 100–500,000 g mol^−1^. Tetrahydrofuran was used as the eluent at a flow rate of 1.0 mL min^−1^, at 30 °C. A differential refractive index detector was used and average molar masses (M_n_ and M_w_), as well as dispersity (Ð), were calculated from a calibration curve based on polystyrene (PS) standards from Polymer Laboratories.

Copolymer composition was determined using ^1^H NMR (Varian Mercury 300 MHz, Varian, Santa Clara, CA, USA). Copolymer samples were dissolved in deuterated chloroform and scanned 32 times at room temperature.

Chemical structures of the copolymers and modified PLA were analyzed by Fourier transform infrared spectroscopy. The FTIR spectra of samples were measured at room temperature with 32 scans at a resolution of 1 cm^−1^ using a Nicolet IZ10 spectrometer (Thermo Fisher Scientific, Waltham, MA, USA) over the wavenumbers ranging from 4000 to 600 cm^−1^.

### 2.5. Extrusion Processing

All samples were dried under vacuum for 12 h at 80 °C, in a vacuum oven, before the melt modification process in order to prevent molecular weight reduction by hydrolysis during extrusion. We processed our synthesized PLA by two extrusion cycles at 190 °C in a co-rotating twin-screw extruder (ZSK25-WLE, W&P, Stuttgart, Germany) at 60 rpm to simulate a recycling process. This biopolyester sample was termed rPLA. The screw diameter of the extruder was 25 mm and the length to diameter ratio (L/D) was 40.

Three blends of 2500 g of PLA with 2 wt% of P(S-co-GMA)-b-PS-1, P(S-co-GMA)-b-PS-2, and P(S-co-GMA)-b-PS-3 polymeric chain extenders, respectively, were fed to the extruder, using a gravimetric device. Then, they were melt processed in the co-rotating twin-screw extruder with an increasing thermal profile, from the hopper to the die section, of 125 °C to 190 °C, at a screw speed of 60 rpm.

The compound granules were then held in an oven for 4 h at 80 °C to avoid moisture regaining before further processing. The corresponding rPLA/P(S-co-GMA)-b-PS blends were denoted as bPLA; bPLA−1, bPLA−2 and bPLA−3, respectively.

### 2.6. Intrinsic Viscosity, Viscosity Average Molecular Weight and Melt Flow Rate Measurements

Measurements of intrinsic viscosity, [η], of rPLA and bPLA were conducted at 25 °C using chloroform as the solvent in a Type I Ubbelohde viscometer. The relative viscosity of a sample was measured using the standard dilution method [49]. Then intrinsic viscosity was calculated from the Solomon and Ciuta single-point method [50,51].

Viscosity average molecular weights (viscometric molar masses), ${\bar{M}}_{v}$, were calculated using the Mark-Houwink equation, expressed in terms of constants reported for poly (l-lactide) [52]:

(1) $$\eta =5.45\;\times \;10^{-4}{\left({\bar{M}}_{w}\;\right)}^{0.73}$$

A Dynisco Melt Flow Indexer (Dynisco, Franklin MA, USA) was used to study the effect of the addition of polymer chain extenders on the melt flow rate (MFR) properties of rPLA. The test was performed according to ASTM D1238 (190 °C/2.16 kg) [53].

### 2.7. Rheological Analyses

The rheological properties of bPLA blends were measured using a controlled stress AR-G2 rheometer (TA Instrument, New Castle, USA) using a parallel-plates geometry, at 190 °C. The diameter of the parallel circular plates was 25 mm, and the space between the plates was 1 mm. Measurements were carried out under a nitrogen gas purge to minimize thermo-oxidative degradation phenomena.

Complex viscosity (η*) and storage modulus (G’) were recorded at a constant strain amplitude of 5 %, as a function of angular frequency, from 0.1 to 100 rad s^−1^.

### 2.8. Thermal Analyses

Differential scanning calorimetry (DSC) thermograms were carried out on a DSC-Q1000 system (TA Instruments, New Castle, DE, USA) under a nitrogen atmosphere. Samples of approximately 5 mg were placed in the aluminum pans and heated from 25 to 200 °C at a heating rate of 10 °C min^−1^ and held for 5 min at that temperature in order to get the same thermal history for all samples. They were then cooled down to 25 °C at a cooling rate of 10 °C/min. A second heating scan was also recorded at a heating rate of 10 °C min^−1^ to 180 °C. The second heating run was used to assess glass transition (T_g_), cold crystallization temperature (T_c_), melting temperature (T_m_) and melting enthalpy (ΔH_m_). Crystallinity percentage was calculated upon the second heating by using the following equation:

(2) $$\mathrm{Xc}=\frac{{\Delta H}_{m}}{{\Delta H}_{m}^{0}}\;\times \;100$$

ΔH_m_ is melting energy, obtained from the DSC analysis, and ΔH°_m_ is the theoretical energy of melting for a 100 % crystalline PLA (93.1 J^.^g^−1^) [54].

## 3. Results and Discussion

### 3.1. Synthesis and Characterization of Reactive Copolymers

**Figure 2:**
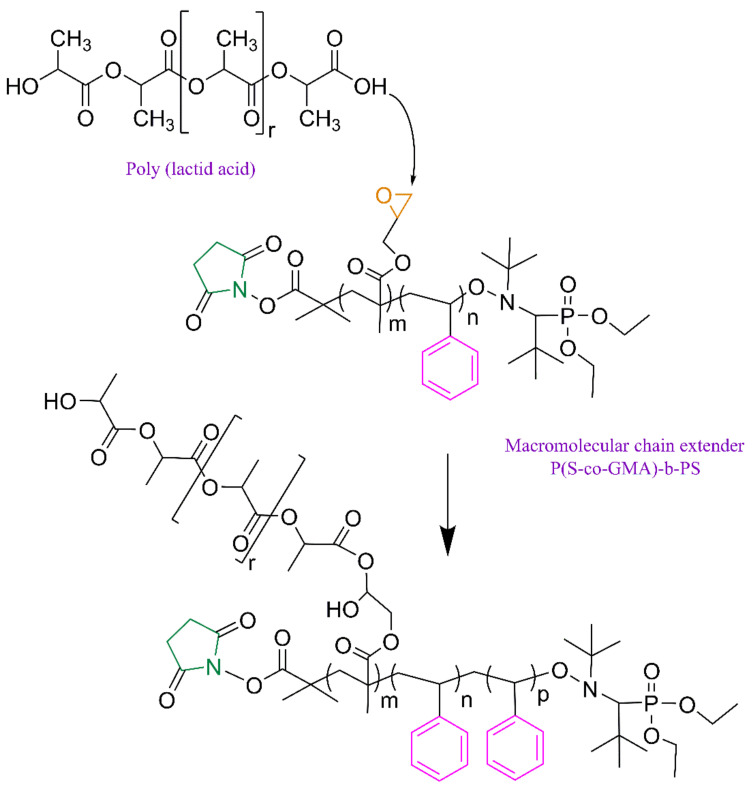
Polymerization scheme for the NMP synthesis of P(S-co-GMA)-b-PS copolymers using NHS-SG1.

The NMP synthesis of the P(S-co-GMA)-b-PS copolymers used in this work is illustrated in Figure 2. Formulations and experimental results for the macro-NMP precursors and P(S-co-GMA)-b-PS copolymers are summarized in Table 1 and Table 2.

**Figure 3:**
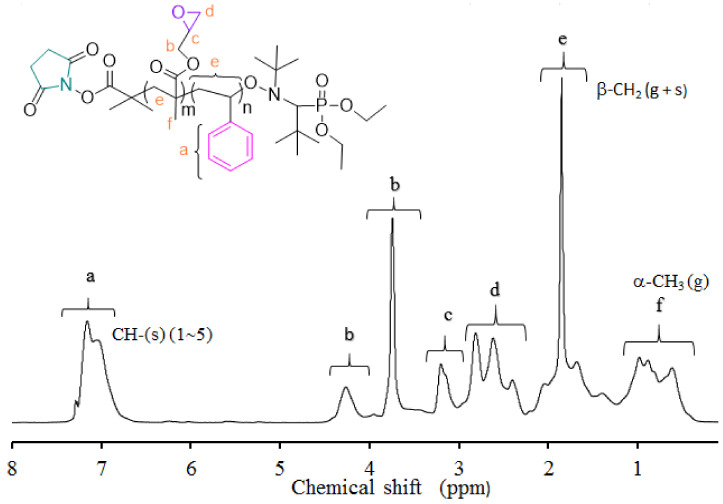
Representative ^1^H-NMR spectrum for P(S-co-GMA) copolymers synthesized by NMP.

Figure 3 shows a representative ^1^HNMR spectrum of P(S-co-GMA) copolymers and assignments of their resonances. Chemical shifts from phenyl protons in the region of 6.6–7.3 ppm, methylene oxy (–OCH_2_–) protons in the region 3.5–4.5, and methyl protons of GMA units in the region 0.5–1.2 ppm are observed. Copolymer composition was determined from ^1^H-NMR characteristic resonances, applying previously reported equations [55]. The synthesized P(S-co-GMA) copolymers contained approximately 41–59 mol% GMA.

**Figure 4:**
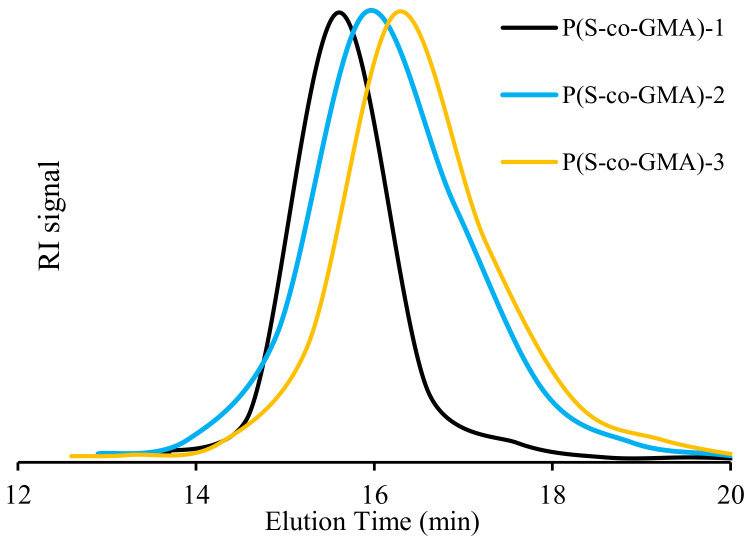
GPC traces for P(S-co-GMA) copolymers synthesized by NMP.

As observed in Figure 4, narrow and monomodal GPC traces were obtained in all the conversion range for the P(S-co-GMA) copolymer samples. It is observed in Table 2 that our NMP-synthesized copolymers presented number average molar masses in the range of 27,000–28,400 g mol^−1^, which is in good agreement with w M_n_,_th_ = 30,000 g mol^−1^, at about 90% conversion. The obtained results suggest that no side reactions occurred during the copolymerization and that it is well-controlled, regarding molar mass development [56,57]. The synthesized P(S-co-GMA) copolymers displayed narrow Ð values, which ranged from 1.22 in the case of 35% GMA, to 1.29 when GMA content is 55%. Ð was somewhat higher for the copolymers richer in GMA, showing that it is more difficult to control the reaction, as GMA content is increased [58].

As explained earlier, the synthesis of block copolymers using the P(S-co-GMA) copolymers just described as macroinitiators for chain-extension reactions were carried out using S at 105 °C. P(S-co-GMA)-b-PS copolymers with conversions around 85% were obtained. The composition of P(S-co-GMA)-b-PS copolymers was determined by nuclear magnetic resonance spectroscopy using the carbons assignments reported in the literature applying previously reported equations [55]. The synthesized P(S-co-GMA)-b-PS copolymers contained approximately 28–46 mol% GMA (see Table 2). Molar mass distribution parameters (M_n_, M_w_, and Ð) for the reactive block copolymers used as chain extenders for rPLA synthesis in this work, at different glycidyl methacrylate compositions, are summarized in Table 2.

### 3.2. Reactive Processing of rPLA/P(S-co-GMA)-b-PS Blends

**Figure 5:**
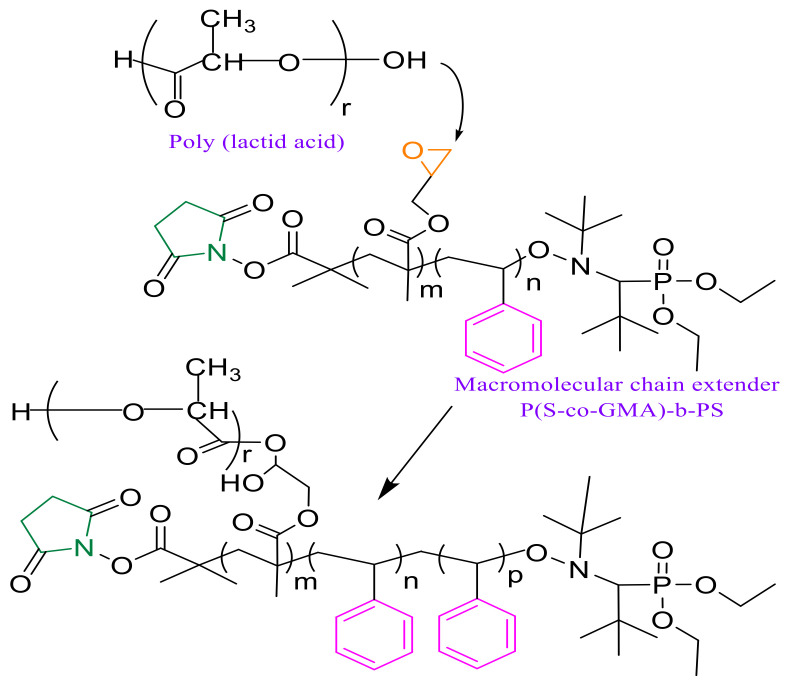
Likely reaction scheme between PLA end groups and P(S-co-GMA)-b-PS epoxy groups.

The proposed chain extension mechanism of PLA with P(S-co-GMA)-b-PS, which involves the reaction between glycidyl groups present in P(S-co-GMA)-b-PS copolymers and PLA carboxyl groups, is shown in Figure 5. According to the literature, the epoxy functional groups from GMA present in P(S-co-GMA)-b-PS can react preferentially with both hydroxyl and carboxyl functional groups present in the biopolymer. However, the reaction with epoxide is more favorable in the case of carboxyl groups [59], resulting in the opening of the epoxy-ring and the creation of new covalent bonds [59,60]. Indeed, the epoxide functionality allows the formation of covalent bonds with the nucleophilic end groups present in the biopolymer. The strong polarization of the hydroxyl group of carboxylic acids ensures fast reactions with epoxy/carboxyl groups, but under suitable conditions the epoxide ring may also react with the weaker hydroxyl nucleophile functionality [61].

**Figure 6:**
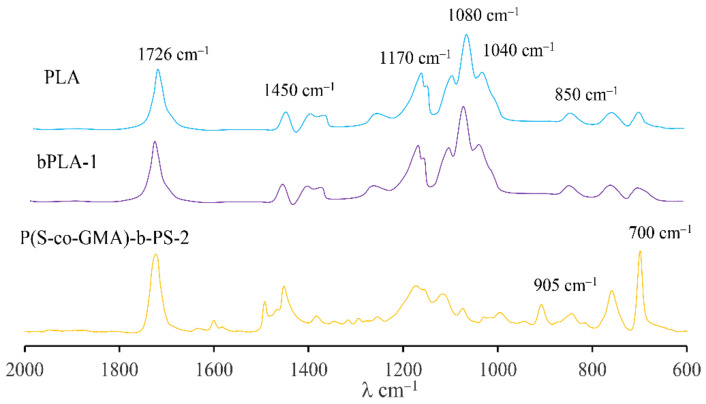
FTIR spectra of PLA, P(S-co-GMA)-b-PS and bPLA.

The Fourier transform infrared spectroscopy (FTIR) spectra of P(S-co-GMA)-b-PS, PLA and bPLA-1 are shown in Figure 6. Several characteristic bands of PLA are observed in the FTIR spectrum of PLA are observed in that figure: The carbonyl group (C= O) stretching vibration appears at 1726 cm^−1^, the C−H deformation vibration band at 1450 cm^−1^, the methyl characteristic band at 1370 cm^−1^. It is also possible to observe the C−O asymmetric stretching band at 1170 cm^−1^ and the C−O−C symmetric band at 1080 cm^−1^. Moreover, in Figure 6 for the FTIR spectrum of P(S-co-GMA)-b-PS, we observed the characteristic bands of the styrene function at 800–600 cm^−1^, and a clear band at 905 cm^−1^, corresponding to the stretching vibration of the epoxy ring.

It is also observed in Figure 6 that the addition of the P(S-co-GMA)-b-PS reactive block copolymer leads to slight changes in the bPLA spectrum, due to the low content of covalent bonds produced by the chain extension reaction.

As observed in Figure 6 for the FTIR spectrum corresponding to bPLA-1, the epoxy ring band associated to the copolymer disappeared in the spectrum of bPLA after the chain extension process of rPLA, and the characteristic band at 700 cm^−1^ related to the bending vibration of C−H out-of-plane of the benzene ring in P(S-co-GMA)-b-PS is clearly observed in the bPLA sample, thus demonstrating the occurrence of chain extension reactions between the recycled biopolymer and the reactive extender copolymer.

### 3.3. Intrinsic Viscosity (IV) Measurements

Progress and performance of the chain extension reaction can be monitored by recording changes in time of [η] and MFR, since longer chains should theoretically increase [η] and reduce MFR.

**Figure 7:**
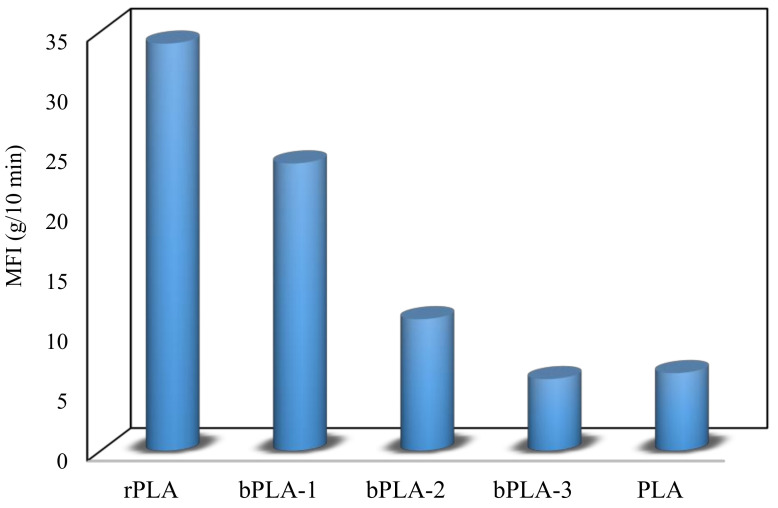
Melt flow rate for rPLA and chain extended rPLA blends.

The melt flow behavior of the unmodified and chain extended rPLA was characterized by MFR measurements (Figure 7). As observed in Figure 7, the MFR of rPLA is 34 g/10 min, a remarkable increase in comparison to PLA, which presents an MFR of 6.5 g/10 min. This increase in MFR may be explained by molecular scission from thermo-mechanical stresses during melt processing, where the biopolyester is exposed to an extra residence time and submitted to increased shear and elongational deformations.

The use of chain extenders decreases MFR, which indicates recovery (increase) of the polymer molar mass. For example, MFR for sample modified bPLA–1 was 24 g/10 min. The best results are obtained when using chain extenders P(S-co-GMA)-b-PS–2, and P(S-co-GMA)-b-PS–3, which correspond to GMA concentrations of 35 and 46, rendering 11 and 6 g/10 min, respectively. This clearly indicates a difference in melt flow behavior between unmodified and chain extended rPLA, which can be attributed to the increased molar mass [62].

**Figure 8:**
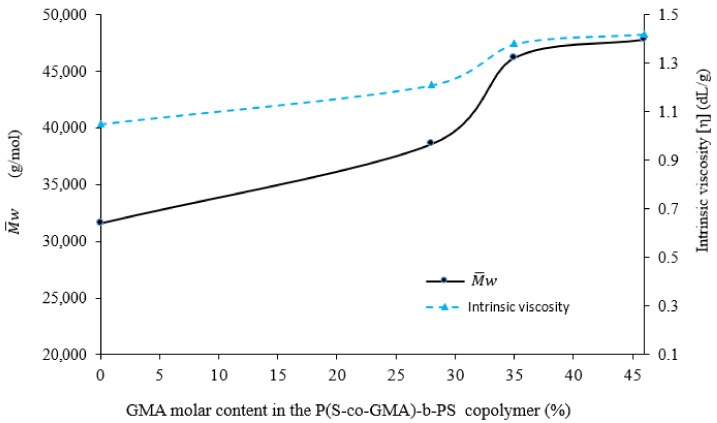
Intrinsic viscosity and ${\bar{M}}_{w}$ for rPLA and bPLA blends.

Intrinsic viscosity, [η], is an important evaluation parameter to assess the action of chain extenders on rPLA since it is related to the molar mass. The values of [η] and ${\bar{M}}_{w}$ for rPLA and rPLA processed with the chain extender in terms of GMA content in the P(S-co-GMA)-b-PS copolymers are shown in Figure 8. It is observed that the extrusion process led to a decrease in the intrinsic viscosity of pristine PLA from 1.43 to 1.05 for rPLA, clearly indicating a reduction in molar mass due to thermal and hydrolytic degradation reactions during melt processing [63].

The incorporation of P(S-co-GMA)-b-PS copolymers into the reacting mass results in an increase in rPLA intrinsic viscosity, due to chain extension, which is proportional to GMA concentration. As shown in Figure 8, GMA concentrations between 0 and 28 wt.% in the chain extender copolymers lead to steep increases in both [η] and ${\bar{M}}_{w}$. At these GMA concentrations, the effect is small. Although the chain extension reaction minimizes the effect of degradation, it does not overcome it completely.

As observed in Figure 8, the best results for chain extension are obtained when P(S-co-GMA)-b-PS–2 or P(S-co-GMA)-b-PS–3 (35 and 46 mol% GMA, and intrinsic viscosities of 1.21–1.42 dL/g, respectively), are used. At these epoxide concentrations, the chain extension reactions seem to trigger the formation of crosslinks and overcome polymer degradation, as indicated by the higher [η] and ${\bar{M}}_{w}$ obtained, compared to those of rPLA.

Higher concentrations of GMA in P(S-co-GMA)-b-PS contribute to increase the probability of reaction between the functional groups (carboxyl, -OH and -COOH groups) present in degraded rPLA and reactive functional groups (epoxide) of the copolymers, leading to chemical bonding of several PLA chains, and to an increase intrinsic viscosity and ${\bar{M}}_{w}$. It is also important to note from the samples with 46 mol% of GMA that their viscosity average molecular weights, ${\bar{M}}_{v}$, values are close to that of pristine-PLA (48,300 g/mol, [η] = 1.43).

### 3.4. Thermal Analyses

**Figure 9:**
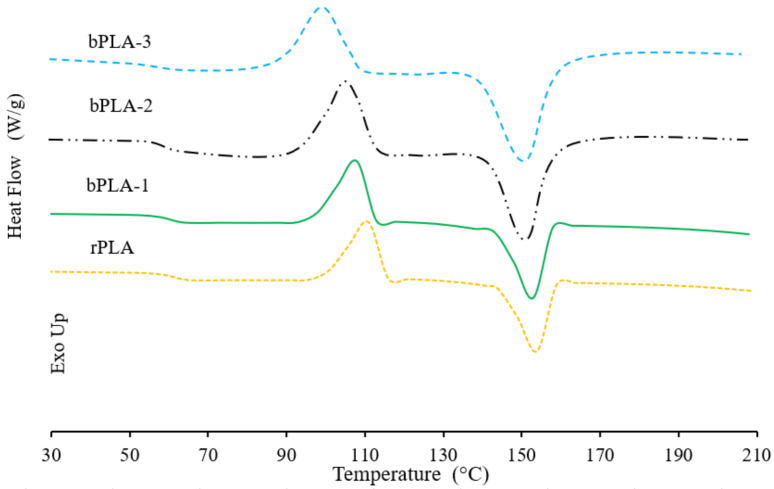
DSC curves for rPLA and bPLA samples during the second heating step. The curves are vertically shifted for easier comparison.

The effects of the chain extension process on the melting and crystallization characteristics of rPLA are shown in Figure 9. During the heating scan, DSC thermograms for rPLA and bPLA show a glass transition (T_g_) around 60 °C. It is observed that the values of T_g_ for rPLA and modified PLA differ negligibly (see also Table 3). An endothermic peak corresponding to the melting of the rPLA crystalline structures is observed at around 150 °C [64]. It is also observed in Figure 9 that this peak decreases slightly when rPLA and bPLA samples are compared, showing that the P(S-co-GMA)-b-PS chain extender may lead to the formation of imperfect crystalline structures. Finally, an exothermic peak which corresponds to the cold crystallization of rPLA is observed at around 110 °C during the heating scan.

Table 3 shows that both recycled PLA and PLA have similar degrees of crystallinity, which indicates that PLA degradation did not have a significant effect on the degree of crystallinity. Similar observations have been reported in the literature [18]. However, there was a slight decrease in cold crystallization temperature (T_cc_) in the recycled material (T_cc_), compared to PLA. This difference is caused by degradation of the biopolymer during the recycling process, which reduces the molar mass of the material. The shorter polymer chains have increased mobility, which allows them to rearrange into crystalline structures and crystallize at lower temperatures [65].

The addition of P(S-co-GMA)-b-PS into rPLA caused a notable decrease in T_cc_, from 111 to 98 °C. Cold crystallization temperature changes to lower values as the molar content of GMA in the P(S-co-GMA)-b-PS chain extender is increased. T_CC_ of sample bPLA-3, which contained higher epoxide content (46 mol% G), decreased to 98 °C, which is indeed significant. These results can be attributed to the nucleating effects from chain extenders segments that induce crystallization, leading to an increase in the level of crystallinity and accelerated cold crystallization, which results in lower T_CC_ [40,66].

Crystallinity has a strong effect on end-use applications of rPLA. It fundamentally depends on molecular structure and crystallization conditions. As observed in Table 3, PLA and rPLA have similar degrees of crystallinity (X_c_) However, rPLA modified with chain extender copolymers shows a higher degree of crystallinity (Xc) than both unmodified rPLA and virgin PLA (PLA).

Our results indicate that crystallinity increases when reactive chain extender copolymers are used. For instance, blending rPLA with P(S-co-GMA)-b-PS–3 increases crystallinity to 32%. High concentrations of chain extender copolymers lead to rPLA molar mass increases, with the corresponding reduction on molecular mobility of polymer chains. However, the nucleating effect of PS blocks in rPLA chains opposes this molar mass trend effect and, as a result, both crystallization rate and biopolymer degree of crystallinity increased [67].

### 3.5. Effect of the Incorporation of Chain Extenders on rPLA Rheological Behavior

**Figure 10:**
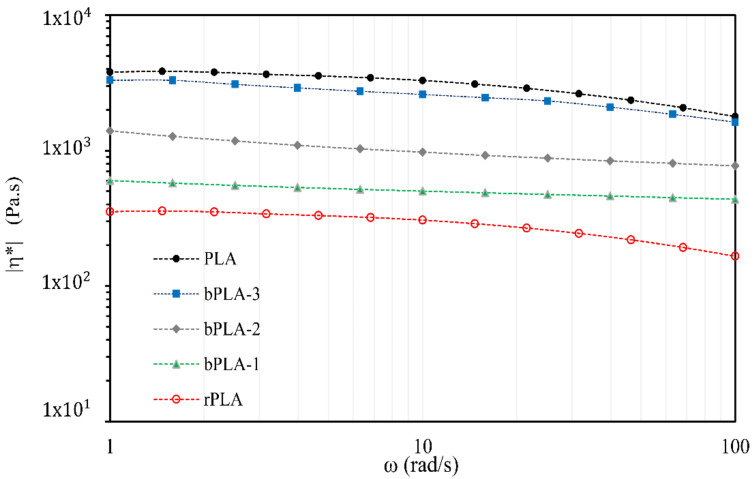
Complex viscosity of rPLA and bPLA blends.

Rheological properties are important to assess biopolymer performance during melt processing. Experimental profiles of complex viscosity (ƞ*) versus angular frecuency (ω) for PLA, rPLA and bPLA blends with different levels of GMA are shown in Figure 10. It is observed that PLA follows a Newtonian behavior in the low-frequency region (<10 rad∙s^−1^) and shear thinning beyond 10 rad∙s^−1^, highlighted by a decrease of the complex viscosity modulus. A large decrease in viscosity for rPLA, from 3700 to 354 Pa∙s, caused by polymer degradation, is clearly observed in Figure 10.

On the other hand, the rheological curves of Figure 10 for all the cases corresponding to chain extended rPLA showed noticeable shear sensitivity behavior, with pronounced shear-thinning responses. This non-Newtonian behavior can be attributed to the introduction of long-chain branches during the reactive extrusion process and to the improvement of melt stability during processing [68]. As observed in Figure 10, the incorporation of P(S-co-GMA)-b-PS chain extenders significantly improves (increase) the complex viscosity of rPLA. Specifically, at a given oscillatory angular frequency, the complex viscosity of bPLA is markedly higher than that of rPLA (~350 Pa∙s at 1 rad∙s^−1^); ƞ* considerably increases as the amount of GMA in P(S-co-GMA)-b-PS is increased. From all the cases analyzed in this contribution, bPLA–3 shows the highest value of complex viscosity (about 3300 Pa∙s at 1 rad∙s^−1^). This improvement in complex viscosity is a second piece of evidence that the chain extension reaction between the reactive groups present in P(S-co-GMA)-b-PS and the ends groups of rPLA took place. This result can be attributed to chain extension and branching, which increased the average molar mass of recycled biopolymer.

**Figure 11:**
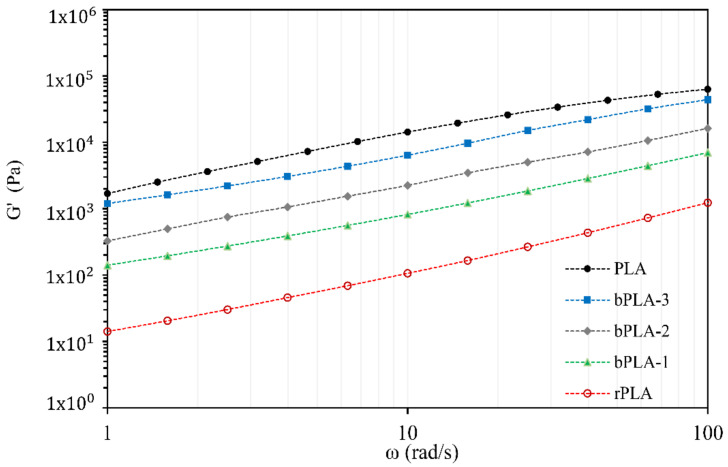
Storage modules G’ vs. frequency for r-PLA and bPLA blends.

Additional confirmation about the rheological performance of modified rPLA after the incorporation of P(S-co-GMA)-b-PS copolymers can be obtained from examination of storage modulus (G’) versus frequency profiles. A plot of storage (G’) modulus of rPLA and bPLA versus frequency is shown in Figure 11. An improvement in melt elasticity, reflected by an increase in G’, is observed in Figure 11. When G’ increases, melt elasticity improves and melt strength also increases. As observed in Figure 11, modulus G’ for all modified rPLA samples is higher than the corresponding G’ values for unmodified rPLA, and they increase progressively with higher epoxide content in the copolymers.

G’ values are particularly higher in the low-frequency region, which indicates that modified rPLA is more elastic than unmodified rPLA. This also indicates that the flexibility of modified rPLA is improved. Low frequencies allow the polymer time for oscillation and responding. Viscous properties become more dominant at lower frequencies, while elastic properties are dominant at higher frequencies [69]. These effects of reactive copolymers on G’ can be explained by chain entanglement and chemical reaction of the end groups in rPLA with epoxy groups from P(S-co-GMA)-b-PS copolymers.

## 4. Conclusions

Poly(styrene-co-glycidyl methacrylate)-block-polystyrene copolymers containing epoxy-functional groups with defined properties, covering a wide range of epoxide content, were synthesized by nitroxide-mediated polymerization and evaluated as chain extenders of rPLA biopolyester.

The incorporation of reactive P(S-co-GMA)-b-PS copolymers during the melt processing of rPLA showed significant improvements in viscosity, melt index and thermal stability. Our results show that increasing epoxide content leads to higher crystallization rates and higher degrees of crystallinity. Chain extension using our NMP-synthesized copolymers caused shifts in the cold crystallization temperature of modified recycled PLA to lower values and higher degrees of crystallinity, compared to unmodified recycled PLA. The crystallinity of rPLA increased from 21% to 32% when the P(S-co-GMA)-b-PS copolymer with the highest GMA content was added to rPLA.

Rheological investigation of modified rPLA blends with P(S-co-GMA)-b-PS copolymers exhibited higher complex viscosities and higher storage modulus indicating that the flexibility of modified rPLA was improved. The improvement in the rheological properties of the recycled biopolymer became more pronounced as the concentration of reactive epoxy functional groups was increased. This behavior is explained by the recombination of rPLA broken chains, leading to increases on average molar masses and chain entanglements.

This contribution demonstrates that effective chain extending action of P(S-co-GMA)-b-PS copolymers during the recycling of PLA is possible. This technological route may contribute to improve the properties and recyclability of PLA with environmental benefits by reducing the consumption of raw materials.

## Figures and Tables

**Table 1:** Experimental conditions for the synthesis of P(S-co-GMA) copolymers at T = 105 °C using N-hydroxysuccinimidyl-functionalized SG1 (NHS-SG1) as NMP controller.

Expt. ID	[GMA]o mol L^−1^	[S]o mol L^−1^	[NHS-SG1] mol L^−1^	f_GMA_^a^	Mn g mol^−1^	Mw g mol^−1^	Ð
P(S-co-GMA)-1	2.490	5.810	0.0320	35	27,000	32,900	1.22
P(S-co-GMA)-2	3.276	4.915	0.3130	45	27,600	34,800	1.26
P(S-co-GMA)-3	4.043	4.043	0.0326	55	28,400	36,600	1.29

^a^ f_GMA_ = Initial feed composition on GMA, mol%, ^a^FGMA = GMA molar content in the copolymer calculated by ^1^H NMR, number average molecular weight (Mn), weight average molecular weight (Mw), and dispersity (Ð) determined by GPC.

**Table 2:** Molecular properties of synthesized P(S-co-GMA)-b-PS copolymers used as polymeric chain extenders of rPLA.

Expt. ID	F_GMA_^a^ mol%	Mn g mol^−1^	Mw g mol^−1^	Ð
P(S-co-GMA)-b-PS-1	28	35,500	46,100	1.30
P(S-co-GMA)-b-PS-2	35	36,300	47,900	1.32
P(S-co-GMA)-b-PS-3	46	37,200	50,600	1.36

^a^ FGMA = GMA molar content in the copolymer calculated by ^1^H NMR, number average molecular weight (M_n_), weight average molecular weight (M_w_), and dispersity (Ð) determined by GPC.

**Table 3:** Thermal properties of rPLA and modified rPLA as function of GMA content.

Sample	Tg (°C)	Tcc (°C)	Tm (°C)	Xc (%)
PLA	60	113	153	19
rPLA	60	111	153	21
bPLA−1	59	108	153	24
bPLA−2	59	105	152	27
bPLA−3	59	98	151	32

## Data Availability

Data is contained within the article.
